# Screening for Cognitive Impairment in Parkinson's Disease: Improving the Diagnostic Utility of the MoCA through Subtest Weighting

**DOI:** 10.1371/journal.pone.0159318

**Published:** 2016-07-20

**Authors:** Sophie Fengler, Josef Kessler, Lars Timmermann, Alexandra Zapf, Saskia Elben, Lars Wojtecki, Oliver Tucha, Elke Kalbe

**Affiliations:** 1 Department of Medical Psychology, Neuropsychology and Gender Studies, University Hospital Cologne, Cologne, Germany; 2 Institute of Gerontology & Center for Neuropsychological Diagnostics and Intervention (CeNDI), University of Vechta, Vechta, Germany; 3 Department of Neurology, University Hospital Cologne, Cologne, Germany; 4 Department of Neurology, Center for Movement Disorders and Neuromodulation, University Hospital Düsseldorf, Düsseldorf, Germany; 5 Department of Clinical and Developmental Neuropsychology, Faculty of Behavioural and Social Sciences, University of Groningen, Groningen, Netherlands; Duke University, UNITED STATES

## Abstract

**Background:**

Given the high prevalence of cognitive impairment in Parkinson’s disease (PD), cognitive screening is important in clinical practice. The Montreal Cognitive Assessment (MoCA) is a frequently used screening test in PD to detect mild cognitive impairment (PD-MCI) and Parkinson’s disease dementia (PD-D). However, the proportion in which the subtests are represented in the MoCA total score does not seem reasonable. We present the development and preliminary evaluation of an empirically based alternative scoring system of the MoCA which aims at increasing the overall diagnostic accuracy.

**Methods:**

In study 1, the MoCA was administered to 40 patients with PD without cognitive impairment (PD-N), PD-MCI, or PD-D, as defined by a comprehensive neuropsychological test battery. The new MoCA scoring algorithm was developed by defining Areas under the Curve (AUC) for MoCA subtests in a Receiver Operating Characteristic (ROC) and by weighting the subtests according to their sensitivities and specificities. In study 2, an independent sample of 24 PD patients (PD-N, PD-MCI, or PD-D) was tested with the MoCA. In both studies, diagnostic accuracy of the original and the new scoring procedure was calculated.

**Results:**

Diagnostic accuracy increased with the new MoCA scoring algorithm. In study 1, the sensitivity to detect cognitive impairment increased from 62.5% to 92%, while specificity decreased only slightly from 77.7% to 73%; in study 2, sensitivity increased from 68.8% to 81.3%, while specificity stayed stable at 75%.

**Conclusion:**

This pilot study demonstrates that the sensitivity of the MoCA can be enhanced substantially by an empirically based weighting procedure and that the proposed scoring algorithm may serve the MoCA’s actual purpose as a screening tool in the detection of cognitive dysfunction in PD patients better than the original scoring of the MoCA. Further research with larger sample sizes is necessary to establish efficacy of the alternate scoring system.

## Introduction

Cognitive dysfunction is frequent in patients with Parkinson’s disease (PD). They are highly relevant, as they limit the patients’ quality of life, increase caregiver burden [1], are an important indication for institutionalization [2], and are related to disease prognosis [3] and mortality [4,5]. Already 20% of unmedicated de-novo PD patients have cognitive symptoms [6,7]. Mild Cognitive Impairment (MCI) affects about 27% of non-demented PD patients (PD-MCI) [8], and about 30% of PD patients develop Parkinson’s disease dementia (PD-D) [9]. Thus, for an optimal management of PD patients, early detection of cognitive symptoms in clinical practice is of utmost importance.

Cognitive screening tests are time-economic and efficient tools to detect cognitive symptoms and dementia [10] and can represent the initial step in a process of clinical decision-making. The Montreal Cognitive Assessment (MoCA) [11] is a widely used tool that has also been recognized to be efficient in detecting cognitive symptoms in PD patients [12–14]. Its sensitivity has consistently been proven to be higher than that of the Mini Mental State Examination (MMSE) [15,16], especially for detecting milder cognitive symptoms [17,18]. The MoCA has also been shown to be suitable to differentiate PD patients with different cognitive states (with no cognitive impairment, mild cognitive impairment, or dementia) from healthy controls [13].The combination of subtests can be regarded as a strength of the MoCA, as a broad range of cognitive domains is covered and assessed with established test paradigms, including very sensitive tasks for the evaluation of executive dysfunction [19] which is of special relevance in PD [20,21]. However, it is striking that its scoring procedure is not based on the discriminant power of the individual subtests. Rather, it is based on the raw scores of the subtests which are simply summed up to a total score of 30 points. This leads to the fact that, for example, the verbal fluency task, although known to be very sensitive in PD patients [22], receives only 1 point (and thus represents only 1/30^th^ of the total score), while, for example, the language items “naming”–a function that is by far less frequently affected in PD—constitute 3/30^th^ of the total score.

Previous research already indicated that the MoCA subtests may differ substantially in their respective diagnostic values. In a study by Ohta et al. [23] including 304 PD patients, three MoCA subtests did not discriminate between patients with low, middle, and high MoCA total scores: The target tapping task, the naming task, and the orientation task. Even though no comprehensive neuropsychological evaluation was used to establish the cognitive state of the participants, this suggests that these items did not add additional information to the determination of the overall cognitive status of the PD patients. In a study [24] focusing on patients with MCI and Alzheimer’s disease (AD), it was shown that only the learning, digit span, serial subtraction, repetition, verbal fluency, abstraction and memory task discriminated between healthy control participants and the MCI group, whereas only the clock drawing task, rhino naming, memory, and orientation tasks discriminated between the MCI and the AD group. Although this study did not include PD patients, it provides further evidence that the MoCA subtests differ considerably in their diagnostic accuracy. Thus, the question arises as to whether an alternative scoring procedure reflecting the subtests’ ability in detecting cognitive dysfunction in PD would increase the instrument’s sensitivity. In fact, scoring procedures of cognitive screening instruments which consider the subtests’ individual discriminant validity and include corresponding weighting of subtests have been proposed [25].

Thus, the aim of this pilot study was to develop an initial version of a new scoring procedure for the MoCA which considers the subtests’ power to detect cognitive symptoms in PD, and to test whether it improves the ability to discriminate PD patients without cognitive impairment (PD-N) from patients with PD-MCI and PD-D. For this purpose, a scoring algorithm was developed and tested in a first sample of PD patients (study 1). Study 2 evaluated the new scoring algorithm on an independent sample of PD patients. In both study samples, the tool’s diagnostic accuracy of the “classical” MoCA and the MoCA with the newly developed scoring system was calculated and compared.

## Methods

### Recruitment of patients and definition of diagnostic groups (PD-N, PD-MCI, PD-D)

In both study 1 and study 2, data from neuropsychological routine testing (for a detailed description please see section “neuropsychological examination” below) of consecutively recruited inpatients with idiopathic PD according to the UK Parkinson’s Disease Society Brain Bank clinical diagnostic criteria [26] seen at the Parkinson’s Disease and Movement Disorders Unit of the Department of Neurology, University Hospital of Cologne, Germany, were included. No limitations with regard to gender, age, race, or disease duration were applied, but only data from patients with German as mother tongue and with no neurological diseases other than PD were included. Exclusion criteria were severe cognitive dysfunction associated with aphasia as well as an overall physical health state which made it impossible to conduct the neuropsychological tests.

Depressive symptoms were assessed with the Geriatric Depression Scale 15 (GDS-15) [27]. Motor symptoms were examined by experienced neurologists with the Unified Parkinson’s Disease Rating Scale (UPDRS) [28]. Total dopaminergic treatment was calculated as the levodopa equivalent daily dose (LEDD) according to the following formula: 1 mg pergolide = 1 mg lisuride = 1 mg pramipexole = 2 mg cabergoline = 5 mg ropinirole = 10 mg bromocriptine = 10 mg apomorphine = 20 mg dihydroergocryptine = 100 mg levodopa proposed by Tomlinson et al. [29].

Patients were classified as PD patients with no cognitive dysfunction (PD-N) or as PD-MCI or PD-D patients according to the Movement Disorder Society (MDS) Task Force Level II criteria for PD-MCI [8] and MDS criteria for PD-D [30], respectively. More specifically, PD-MCI criteria included 1) a diagnosis of PD according to Queen Square Brain Bank criteria [26], 2) cognitive complaints reported by the patient or observed by the clinician, 3) deficits on at least two neuropsychological tests, either two impaired tests in one domain, or one impaired test in two different domains, and 4) no dysfunction of activities of daily living (ADL) because of cognitive impairment. To assess the latter point, subjective reports of cognitive decline and ADL impairments due to cognitive dysfunction were obtained during a semi-structured interview preceding the neuropsychological examination. Patients were asked to indicate whether they had noted any meaningful change over the course of their illness in their ability to remember things, to pay attention, to plan and execute complex activities, their visuospatial functions, or their language functions. If subjective impairments were reported, patients were asked to evaluate whether these deficits caused any ADL impairment, independent of the impairment ascribable to the motor symptoms. MDS PD-D criteria [30] included 1) a diagnosis of PD according to Queen Square Brain Bank criteria, 2) cognitive deficits in at least two of the five cognitive domains named above as evidenced by deficits in at least one test for a domain, and 3) ADL impairments that are not primarily attributable to motor symptoms associated with PD, assessed as described above. The ethics commission of the Faculty of Medicine of the University Hospital of Cologne advised that no formal approval was required as the study only involved retrospective analysis of data collected during standard care and no identifying information is reported.

### Neuropsychological examination

The neuropsychological examination was conducted by an experienced neuropsychologist (sf) during the patients’ on-phase after medication and, in case of deep brain stimulation of the subthalamic nucleus (STN-DBS), with the deep brain stimulator switched on. In addition to the MoCA, the cognitive state was measured using the MMSE [15,16]. Both screening procedures were not used for classification of PD-N, PD-MCI, and PD-D. In accordance with MDS task force criteria for PD-MCI and PD-D, each cognitive domain was assessed with two neuropsychological tests: *Verbal Memory* was measured with the word list memory and the word list recall task of the Consortium to Establish a Registry for Alzheimer's Disease test battery (CERAD) [31]. *Executive functions* were assessed with the Trail Making Test (TMT) B/A Index and the semantic verbal fluency task (animal naming) of the CERAD. *Attention* was tested with the Wechsler Memory Scale—Revised (WMS-R) [32] block span task and the WMS-R digit span backward task. *Visuospatial functions* were measured with the mental rotation test of the Achievement Testing System 50+ (LPS 50+) [33], and the CERAD constructional praxis task. Finally, *language* was tested with the CERAD 15-item Boston Naming Test and the auditory comprehension subtest of the Aphasia Check List (ACL) [34]. For the CERAD subtests, a score of ≥ 1.128 standard deviations below the normative data was considered to represent a deficit, as recommended in the German CERAD test manual [35]. This cutoff score corresponds to a percentile rank of ≤ 10 for the WMS-R subtests and the mental rotation subtest of the LPS 50+. Age-adjusted scores were used for all tests. Furthermore, CERAD scores were also gender- and education-corrected.

### Statistical analyses

All data were completely anonymized prior to analysis (ek). They were analyzed using IBM SPSS Statistics 22. In both studies, possible differences between demographic, clinical and neuropsychological test data of the three diagnostic patient groups were examined by means of a Univariate Between-Subjects ANOVA. Post-hoc comparisons for the neuropsychological variables were made using Bonferroni correction. Results of the Shapiro-Wilk test of normality showed that not all clinical and sociodemographic variables were normally distributed. For these variables, the non-parametric equivalent Kruskal Wallis-Test and the Mann-Whitney U test for pairwise comparisons (with Bonferroni adjusted alpha-levels for the neuropsychological test data) were used. The Fisher’s Exact test was used for dichotomous variables. A one-way Between-Subjects ANOVA was conducted to compare MoCA Total scores between impaired (PD-MCI and PD-D) and unimpaired patients (PD-N). For the comparison of neuropsychological test data and of the original and weighted MoCA scores, the effect size *r* was computed. For non-parametric tests, scores were transformed into ranks, partial eta-squared was computed by running an ANOVA on the ranked scores, and finally the square root was taken to obtain *r*. In both studies, it was tested whether the new weighting was successful in increasing the overall discriminating power of the MoCA. For this purpose, the diagnostic accuracy of the original and the new weighted total score were compared by means of their sensitivity and specificity as well as the Youden’s Index [36] [= sensitivity–(1 –specificity)] which is a frequently used summary measure of the ROC curve that captures the performance of a diagnostic test with a single numerical value. For a test with poor diagnostic accuracy, Youden's index equals 0, and in a perfect test Youden's index equals 1. Furthermore, the positive predictive value and the negative predictive value of the original and the weighted MoCA score were calculated for study 1 and 2. The AUC values of the original and the weighted MoCA were compared using the method of de Long et al. [37]

#### Weighting and new scoring procedure of the MoCA

**Fig 1:**
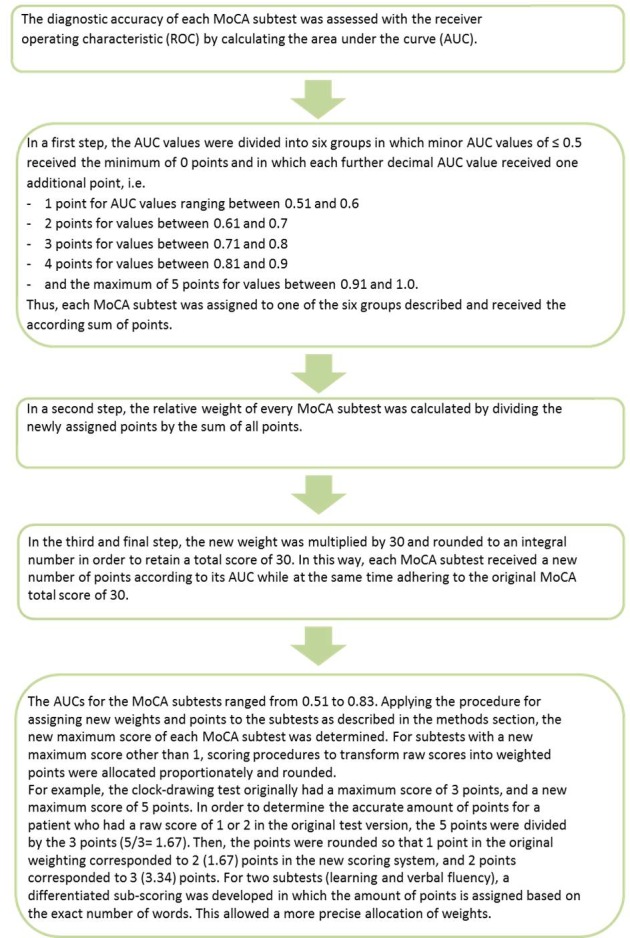
Presentation of the development of the new MoCA weighting.

In the current analysis, the AUCs of the MoCA were calculated as a measure of the discriminating power of the weighted MoCA algorithm, since the MoCA only proposes one cutoff score to discriminate cognitively unimpaired versus cognitively impaired patients, without distinction between MCI and dementia [11]. Therefore, in the determination of sensitivity and specificity, PD-MCI and PD-D patients were analyzed together as a group of patients with cognitive impairment. Fig 1 shows the development process of the new weighting system.

The weights of the MoCA subtests in the original version, the AUCs for each subtest, new weights and new maximum points are displayed in Table 1. Table 2 shows the scoring algorithm for assigning raw scores to weighted points for each subtest. In S1 Fig, a case report of a re-scored MoCA is shown.

**Table 1:** AUCs of the MoCA subtests in study 1, original and weighted subtest points.

Domain*	Subtests	Original Points	% Original Points	AUC	Weighted Points	% Weighted Points
Visuospatial and executive functions	Alternating Trail Making	1	3	0.74	4	13
	Cube copy	1	3	0.67	3	10
	Clock-drawing	3	10	0.83	5	17
Naming	Animal Naming	3	10	0.56	1	4
Attention	Digit Span	2	7	0.59	1	4
	Target tapping	1	3	0.58	1	4
	Serial subtraction	3	10	0.51	1	4
Language	Repetition	2	7	0.66	3	9
	Verbal fluency	1	3	0.62	3	9
Abstraction	Abstraction	2	7	0.52	1	4
Memory	Learning	0	0	0.62	3	9
	Memory	5	17	0.61	3	9
Orientation	Orientation	6	20	0.56	1	4

* as assigned by the authors of the MoCA [11].

**Table 2:** Conversion table for scoring of the MoCA subtests.

	MoCA Subtest	Original Points	New Points
Visuospatial and executive functions	Alternating Trail Making	0	0
		1	4
	Cube copy	0	0
		1	3
	Clock-drawing	0	0
		1	2
		2	3
		3	5
Naming	Animal Naming	0	0
		1	0
		2	1
		3	1
Attention	Digit Span	0	0
		1	0
		2	1
	Target tapping	0	0
		1	1
	Serial subtraction	0	0
		1	0
		2	0
		3	1
Language	Repetition	0	0
		1	2
		2	3
	Verbal fluency	0	0 to 3 words: 0
		1	4 to 7 words: 1
			8 to 10 words: 2
			> 10 words: 3
Abstraction	Abstraction	0	0
		1	0
		2	1
Memory	Learning	0	3 to 5 words: 1
			6 to 8 words: 2
			9 to 10 words: 3
	Memory	0	0
		1	1
		2	1
		3	2
		4	2
		5	3
Orientation	Orientation	0	0
		1	0
		2	0
		3	0
		4	1
		5	1
		6	1

## Results

### Study 1

#### Study sample

Forty PD patients were consecutively recruited between April and June 2013, 15 of which had no cognitive impairment (PD-N), 14 had PD-MCI, and 11 were classified as having PD-D. Mean age was 65.4 years (*SD* 9.8; range 46 to 86 years), mean education (school and higher education) was 14.4 years (SD 3.8, range 7 to 21 years), and 30% (n = 12) of the sample was female. Eleven participants scored above four points on the GDS-15 suggesting depressive symptoms. Mean UPDRS score was 24 (SD 12.8), and the mean LEDD in the sample was 460.4 (SD 410.1). Nineteen patients had undergone a STN-DBS. Sociodemographic and clinical data of the three diagnostic groups are shown in Table 3. All neuropsychological test results and group comparisons are shown in the S1 Table.

**Table 3:** Demographic and clinical characteristics of all diagnostic groups in study 1.

		PD-N	PD-MCI	PD-D	*p*-value	PD-cognitively impaired	*p-*value (PD-N vs. PD-cognitively impaired)
		(n = 15)	(n = 14)	(n = 11)		(PD-MCI& PD-D; n = 25)	
Male/female ^a^	n (%)	14 /1	6/8	8/3	.012 ^b^	14/11	.015
Age (years) ^c^	Mean (SD)	63.5 (8.8)	64.2 (9.5)	69.4 (11.1)	.285	66.5 (10.4)	.363
	Median (IQR)^d^	65 (55–68)	61.5 (56.5–73,25)	71 (61–76)		69 (58.5–74.5)	
Education (years) ^d^	Mean (SD)	15.2 (4.1)	13.6 (3.3)	14.2 (4.0)		13.8 (3.5)	
	Median (IQR)	16 (11–18)	13 (11–15.75)	13 (11–18)	.662	13 (11–18)	.455
PD duration ^c^	Mean (SD)	8.4 (4.7)	10.5 ± 5.1	10.6 ± 7.0	.515	10.5 (5.9)	.246
	Median (IQR)	10 (5–12)	10 (7.5–15)	10 (8–14)		10 (8–15)	
Age at diagnosis ^c^	Mean (SD)	55.1 (8.9)	52.9(11.3)	58.7 ± 10.8	.385	55.5 (11.2)	.906
	Median (IQR)	52.0 (51–62)	54 (46–62.5)	60 (49–71)		55.5 (47.25–64.5)	
UPDRS III ^c^	Mean (SD)	18.4 (12.1)	23.9 (11.3)	28.2 ± 11.6	.151	26.1 (11.4)	.078
	Median (IQR)	17.5 (12–24.25)	20 (16–34)	31 (20–35)		30 (16.75–34.25)	
History of DBS ^a^	(% yes)	60%	43%	36%	.526	40%	.333
Interval since DBS	Mean (SD)	21.3 (16.3)	23.8 (16.4)	44.3 (31.6)		32.0 (24.2)	
Implantation (months) ^d^							
	Median (IQR)	17 (5–34)	25 (5–39)	40 (16–76)	.652	26 (11.25–47.25)	.497
LEDD (mg/d) ^c^	Mean (SD)	619.2 (484.6)	429 ± 265.5	396.4 ± 547.6	.194	422.7 (415.4)	.448
	Median (IQR)	448.0 (140–746)	439 (234-25-739.6)	209 (133–400)		300 (199.75–560)	
GDS-15 score ^d^	Mean (SD)	2 (1.7)	3.9 (2.6)	5.9 (3.4)		4.6 (3.0)	
	Median (IQR)	2 (0–3)	4 (1.75–6)	4 (4–9)	.01^e^	4 (2–6.25)	.005
MMSE ^d^	Mean (SD)	29.0 (0.9)	28.3 (1.3)	27.1 (1.8)		27.9 (1.5)	
	Median (IQR)	29 (28.5–30)	29 (27.5–29)	28 (25–29)	.029 ^f^	28.5 (26.25–29)	.036

SD, Standard deviation; IQR, Interquartile range; LEDD, total daily levodopa equivalent dose.

^a^ Fisher`s Exact test was used for dichotomous variables.

^b^ Pairwise comparisons showed that the male/female ratio was higher in PD-MCI compared to PD-N; PD-N = PD-D; PD-MCI = PD-D.

^c^ A univariate Between-Subjects ANOVA with post-hoc comparisons was used for variables that were normally distributed. P-values are shown for the mean. Median and interquartile range are also reported for comparison purposes.

^d^ The Kruskal-Wallis test was used for variables that were not normally distributed. P-values are shown for the median. Groups were compared using the Mann-Whitney U test. Means and standard deviations are also reported for comparison purposes.

^e^ Pairwise comparisons showed that PD-N = PD-MCI = PD-D; PD-N<PD-D.

^f^ Pairwise comparisons showed that PD-N = PD-MCI = PD-D; PD-N>PD-D.

#### Testing the new scoring algorithm of the MoCA in the original sample—Comparison of original and weighted total scores of the MoCA

**Fig 2:**
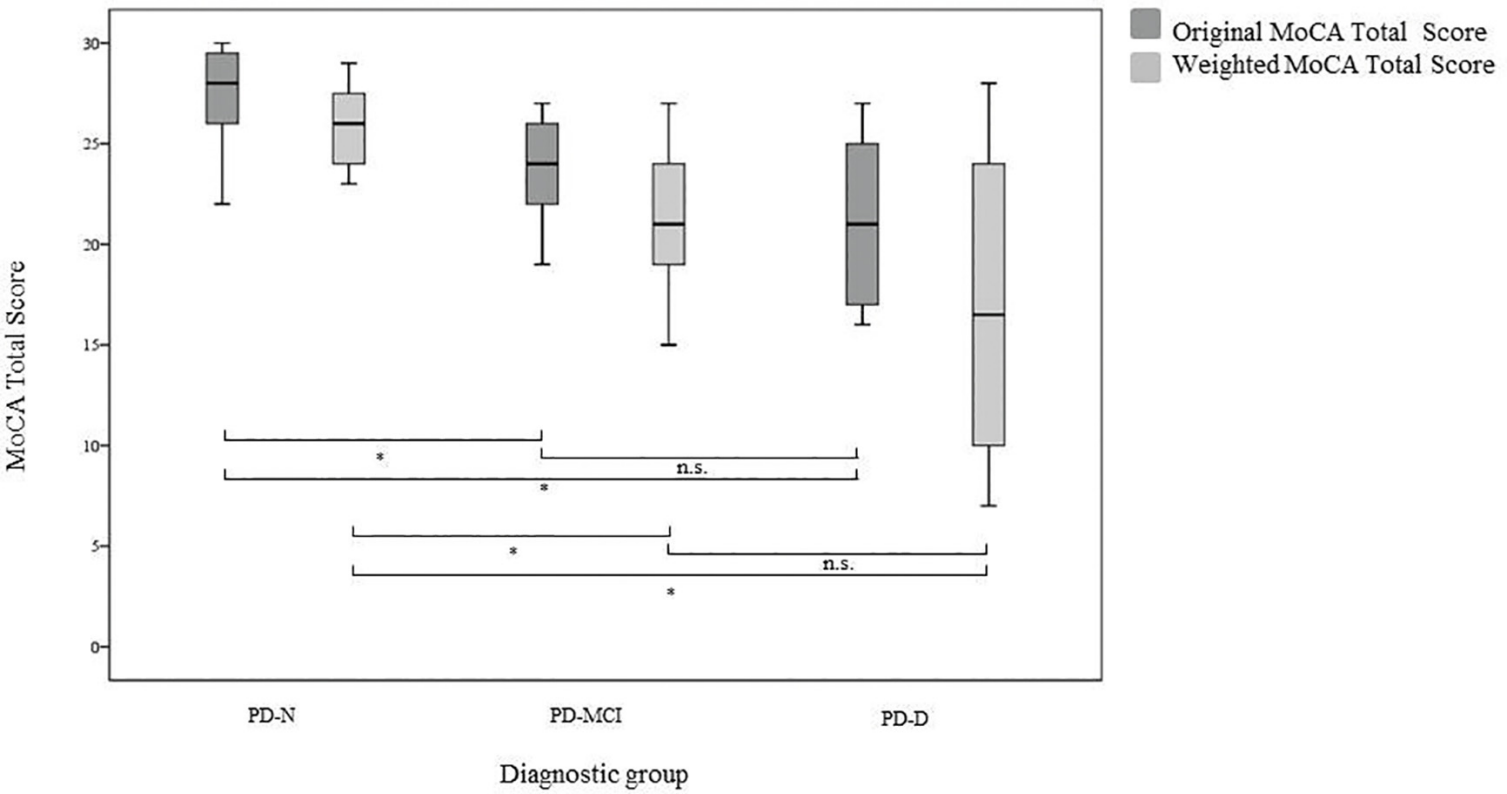
MoCA original and weighted scores by diagnostic group in study 1.

The original and weighted MoCA total scores are displayed in Fig 2. On a descriptive level, all weighted MoCA total scores, especially those in the PD-MCI and the PD-D group, were lower than the original total scores. Both the original and the weighted MoCA total scores differed significantly between the impaired PD patients (PD-MCI and PD-D together) and the unimpaired PD patients (PD-N; F_1,38_ = 8.32, *p* = .006, *r* = .42 and *F*_1,38_ = 9.82, p = .003, *r* = .45, respectively). After excluding PD-D patients, the difference in the MoCA total score between the PD-N and the PD-MCI group was still significant in both versions (*F*_1,27_ = 5.47, *p* = .027, *r* = .41 and *F*_1,27_ = 5.54, *p* = .026, *r* = .41, respectively). As expected, also scores between the PD-N and the PD-D group were significant (*F*_1,24_ = 8.71, *p* = .007, *r* = .52 and *F*_1,24_ = 9.48, *p* = .005, *r* = .53, respectively). For the original and weighted scoring, no significant difference was found between the total scores of PD-MCI and the PD-D patients (*F*_1,23_ = 0.98, *p* = .33, *r* = .20 and *F*_1,31_ = 1.12, *p* = .29, *r* = .22, respectively).

#### Discriminating patient groups with the original and the weighted MoCA total scores

Sensitivities and specificities of the original and the weighted MoCA score were compared using the original cutoff score of 26/30 for cognitive impairment. Analyses showed that the original MoCA total score discriminated patients with preserved cognition (PD-N) from those with cognitive impairment (PD-MCI and PD-D) with a sensitivity of 62.5% and a specificity of 77.7%, while the weighted MoCA total score had an increased sensitivity of 92% and a slightly reduced specificity of 73%. These data correspond to a Youden’s Index of the original and the weighted MoCA total scores of 0.41 and 0.65, respectively. With regard to detection of PD-MCI in contrast to PD-N patients, the original MoCA total score had a sensitivity of 55.5% and a specificity of 83.3%, while the weighted MoCA had a much higher sensitivity of 92.9 and a reduced specificity of 72%. The corresponding Youden’s Indices were 0.33 and 0.64, respectively. Finally, with regard to detection of dementia (PD-N versus PD-D), the original MoCA total score had a sensitivity of 72.8% and a specificity of 83.3%, while the weighted MoCA had a higher sensitivity of 90.9% and a slightly lower specificity of 79%. These data correspond to a Youden’s Index of 0.56 and 0.69, respectively. The PPV of the original MoCA for the detection of any cognitive disorder was 75, and the NPV was 52.6. The PPV of the new MoCA increased to 88.5%, and the NPV increased to 77.8%.

### Study 2

#### Study sample

Twenty-four PD patients were recruited consecutively between November 2014 and February 2015–8 with PD-N, 10 with PD-MCI, and 6 with PD-D, and were included in study 2. Mean age was 65.1 years (*SD* 10.4; range 46 to 78 years), mean education was 12.8 years (*SD* 3.4, range 7 to 20 years), and 21% (n = 5) of the sample was female. Eleven participants scored above four points on the GDS-15 indicating at least mild depressive symptoms. Mean UPDRS score was 20.2 (*SD* 6.2), and the mean LEDD in the sample was 613.8 (SD 354.8). Sixteen patients had undergone STN-DBS. Sociodemographic and clinical data of the three groups are shown in Table 4. Results and comparisons of neuropsychological data of the three groups are shown in the S2 Table.

**Table 4:** Demographic and clinical characteristics of all diagnostic groups in study 2.

		PD-N	PD-MCI	PD-D	*p*-value	PD-cognitively impaired	*p-*value (PD-N vs. PD-cognitively impaired)
		(n = 8)	(n = 10)	(n = 6)		(PD-MCI& PD-D; n = 16)	
Male/female ^a^	n (%)	7/1	9/1	3/3	.161	12/4	.613
Age (years) ^b^	Mean (SD)	66.5 (6.7)	65.0 (10.2)	63.3 (15.5)	.864	64.4 (12.0)	.647
	Median (IQR)	64.5 (62.3–72.3)	64.5 (55.5–74.3)	68.5 (51.8–75.0)		64.5 (57–75)	
Education (years) ^b^	Mean (SD)	14.0 (4.6)	12.9 (2.3)	11.0 (1.6)	.277	12.2 (2.7)	
	Median (IQR)	15 (9.8–18.0)	12 (12.0–13.0)	12 (9.0–12.0)		12 (12–12)	.383
PD duration ^b^	Mean (SD)	7.6 (4.5)	6.4 (3.5)	6.5 (4.7)	.815	6.4 (3.9)	.519
	Median (IQR)	8 (3.3–11.3)	6.5 (4.2–9.0)	6 (4.0–10.3)		6.5 (2.5–9)	
Age at diagnosis ^b^	Mean (SD)	59.0 (8.6)	58.7 (12.75)	57.3 (14.3)	.964	58.19 (12.9)	.874
	Median (IQR)	61 (50.3–65.0)	58.5 (48.8–68.0)	60.5 (47.0–67.3)		60.5 (50.5–65)	
UPDRS III ^b^	Mean (SD)	22.3 (9.6)	18.6 (3.3)	20.0 (6.0)	.894	19.1 (4.1)	.435
	Median (IQR)	17.5 (10–17.5)	20.0 (16.5–22)	20.0 (14–20)		19.0 (15.25–22.0)	
History of DBS ^a^	(% yes)	50%	30%	17% ^d^	.556	25%	.363
Interval since DBS Implantation(months)^c^	Mean (SD)	13.3 (7.9)	21.0 (7.9)	20.0 ^d^		20.8 (6.5)	
	Median (IQR)	13 (5–21)	18 (15–18)	20.0 ^d^	.419	19.0 (15.75–27.5)	.200
LEDD (mg/d) ^b^	Mean (SD)	655.9 (367.5)	682.5 (352.0)	381.3 (329.1)	.355	415.7 (303.7)	.402
	Median (IQR)	635.8 (383.8–1014.58)	747.9 (491.0–939.3)	335.0 (90.0–718.8)		512.2 (314.5–715.9)	
GDS-15 score ^b^	Mean (SD)	2.6 (1.9)	4.9 (3.6)	7.7 (4.9)	.032 ^e^	5.9 (3.9)	.034
	Median (IQR)	3 (0.5–4.0)	5.0 (2.3–7.0)	9.5 (2.8–11)		5 (3–10)	
MMSE ^c^	Mean (SD)	29.25 (0.46)	28.0 (1.56)	26.0 (2.53)		27.25 (2.15)	
	Median (IQR)	29 (29.0–29.75)	28.5 (2)	26 (23.8–27.8)	0.021 ^f^	27.5 (25.25–29.0)	.019

SD, Standard deviation; IQR, Interquartile range; LEDD, total daily levodopa equivalent dose.

^a^ Fisher`s Exact test was used for dichotomous variables.

^b^ A univariate Between-Subjects ANOVA with post-hoc comparisons was used for variables that were normally distributed. P-values are shown for the mean. Median and interquartile range are also reported for comparison purposes.

^c^ The Kruskal-Wallis test was used for variables that were not normally distributed. Groups were compared using the Mann-Whitney U test. P-values are shown for the median. Means and standard deviations are also reported for comparison purposes.

^d^ Only 1 patient in the PD-D group had a history of DBS.

^e^ Pairwise comparisons showed that PD-N = PD-MCI = PD-D; PD<PD-D.

^f^ Pairwise comparisons showed that PD-N = PD-MCI = PD-D; PD-N>PD-D.

#### Testing the new scoring algorithm of the MoCA in an independent study sample—Comparison of original and weighted total scores of the MoCA

**Fig 3:**
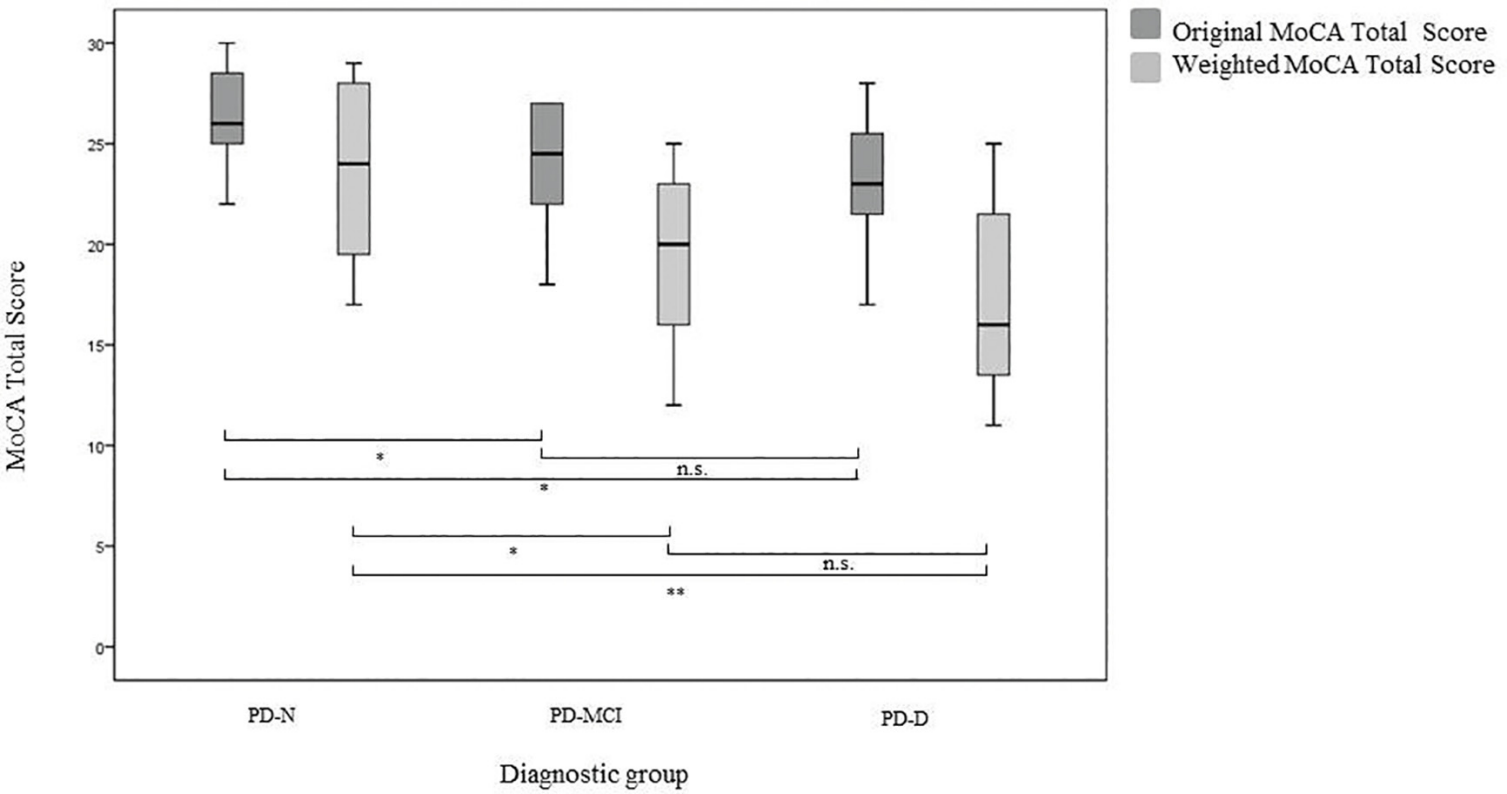
MoCA original and weighted scores by diagnostic group in study 2.

The original and weighted MoCA total scores of study sample 2 are displayed in Fig 3. As in study 1, all weighted MoCA total scores were lower than the original total scores. The original as well as the weighted MoCA scores differed significantly between the impaired PD patients (PD-MCI and PD-D together) and the unimpaired PD patients (PD-N; F_1,22_ = 9.84, *p* = .005, *r* = .56 and *F*_1,22_ = 5.49, p = .02, *r* = .48, respectively). After excluding PD-D patients, the difference in the MoCA total score between the PD-N and the PD-MCI group was still significant for both scoring systems (*F*_1,16_ = 8.15, *p* = .01, *r* = .58 and *F*_1,16_ = 5.29, *p* = .03, *r* = .50, respectively). Scores between the PD-N and the PD-D group were also significant (*F*_1,12_ = 9.75, *p* = .009, *r* = .67 and *F*_1,12_ = 5.67, *p* = .035, *r* = .57, respectively). For both the original and the weighted MoCA, no significant difference was found between the total scores of the PD-MCI and the PD-D patients (*F*_1,14_ = 2.14, *p* = .16, *r* = .36 and *F*_1,14_ = 1.77, *p* = .21, *r* = .33, respectively).

#### Discriminating patient groups with the original and the weighted MoCA total scores

The AUC values of the original and the new MoCA total score were not significantly different (0.87 and 0.83, respectively; *p* = 125). However, considering the individual diagnostic parameters, results indicate that when retaining the original cutoff score of 26/30 points, the original MoCA discriminated patients with normal cognition from those with impaired cognition with a sensitivity of 68.8% and a specificity of 75% whereas the new weighted total score reached a sensitivity of 81.3%, while the specificity remained unchanged with 75%. These values correspond to a Youden’s Index of 0.43 and 0.56, respectively. The original MoCA score had a PPV of 84.6% and a NPV of 54.6%, while the score based on the new algorithm had a PPV of 86.7% and a NPV of 66.7%.

## Discussion

The present study was designed to propose a new scoring algorithm for the cognitive screening instrument MoCA in which the tool’s subtests are weighted according to their sensitivity and specificity and to preliminarily evaluate whether this scoring procedure may improve the overall discriminant value of the screening for the detection of PD-MCI or PD-D. Our data analysis revealed that the new weighted MoCA total score with a cutoff score as proposed by the authors of the test [11] which is widely used in clinical and research settings yields a higher sensitivity than the original MoCA total score at a comparable specificity and that it is thus able to better discriminate cognitively preserved from cognitively impaired PD patients. This result was found both in the original dataset and the validation dataset. More precisely, in the original data of study 1, the sensitivity of the MoCA increased substantially from 62.5 to 92% for the detection of any cognitive disorder (PD-MCI or PD-D), while the specificity only slightly decreased from 77.7 to 73%. Moreover, even in the independent sample of PD patients of study 2, the sensitivity of the MoCA increased from 68.8% to 81.3% while maintaining a specificity of 75%. This means that more cognitively impaired subjects were detected in both patient samples, leading to a decreased number of false negatives. This can be explained by the fact that the relatively easy items, for which the probability of a correct response is very high, became less influential in the new weighting. Consequently, those patients that are indeed cognitively impaired no longer receive numerous points for very easy items that lack a high diagnostic value. In fact, deficits in relevant domains are no longer “masked” or hidden within the total score of the MoCA, so that cognitive dysfunction is more easily detected.

The sensitivity of the original MoCA score in our data (62.5% in study 1 and 68.8% in study 2) is remarkably low. Other studies found that the MoCA in its original form had a sensitivity of 90% [13,14] for PD patients with cognitive impairment. However, a closer look to the study by Hoops et al. [14] reveals that a cutoff score of 26/27 for cognitive impairment was used in their sample of 132 PD patients. Thus, this cutoff is 1 point above the one in our study which corresponds to the one that is proposed in the original test form and which is commonly used in clinical practice. Importantly, when the cutoff score in our study 1 is raised by 1 point, we also achieve a substantially higher sensitivity of 80.6% (which is in line with the result that the AUC values in the two MoCA versions did not differ significantly as AUC values reflect sensitivities and specificities of different cutoff scores). However, at the same time, specificity drops down to 66.7% which approximates the even lower specificity of 53% reported by Hoops et al. [14]. Although the primary goal of a screening test is to detect cognitive impairment, too many false positives should be avoided. Rather, a good balance of sensitivity and specificity should be pursued and is of great clinical use. This is especially important as in clinical practice, screening tests are (unfortunately) often the only tool used—without further elaborate testing. With regard to the sensitivity of 90% for the detection of PD-MCI and PD-D combined and the specificity of 75% reported by Dalrymple-Alford et al. [13], it has to be noted that their participants were cognitively more impaired than the participants in our study: while the mean MoCA score in the original, unweighted version was 23.2 ± 2.5 for PD-MCI and 16.9 ± 4.0 for PD-D in their study, it was considerably higher in our study with values of 23.9 ± 3.2 for PD-MCI and 22.45 ± 4.2 for PD-D. Thus, their deficits were naturally easier to detect. Although the specificity of the MoCA with the weighted scoring system only decreased slightly from 77.7 to 73% in our study 1 (and even remained stable at 75% in study 2), this minor increase of patients who did not suffer from cognitive dysfunction but were classified as cognitively impaired should be commented on. In the new scoring algorithm, the comparably difficult items have a stronger influence on the new total score. The result of this change is that if a patient with preserved cognition (or more accurately: cognitive function with scores above the defined cutoff score as tested in the elaborate neuropsychological test battery) failed one of those more difficult subtests, he or she more easily scored below the cutoff score. The probability that a patient fails a difficult item is naturally higher than for an easy item, and the impact is higher in the weighted MoCA. As a consequence, the test loses specificity. However, as indicated above, the main objective of a cognitive screening instrument is its sensitivity, i.e. to detect affected individuals, while still showing an acceptable specificity. With a high sensitivity of above 80% and a specificity of above 70% in both studies, we conclude that these criteria are fulfilled with the new MoCA scoring scheme.

As expected, our data analysis showed that the subtests differ markedly in their individual predictive values. With regard to the specific subtests which are weighted stronger in the new MoCA scoring procedure on the basis of these predictive values, our results correspond largely to findings concerning the typical cognitive deficits associated with PD. The alternating trail making subtest as an executive task and the clock-drawing subtest as an executive and visuospatial task were the subtests with the highest predictive value, followed by the cube copy, again as a visuospatial task, verbal fluency, again an executive task, and the memory subtests learning and memory. In line with these findings, executive functions are the functions that are most frequently impaired in the early stages of PD [38,39], with verbal fluency as a frequently used and affected task [22], and visuoconstructive deficits are also common [40,41]. Furthermore, memory dysfunction is frequent [42,43]. The high weight that the subtests received through the new weighting is thus meaningful. However, also the subtest repetition of sentences has received a high weight. As this is a classified language task, this seems astonishing at first sight. However, repetition of sentences strongly depends on working memory so that it can be assumed that the reason why PD patients score low on this subtest is dysfunction in working memory, rather than a primary language deficit. Working memory is usually conceptualized as an important subfunction of executive functions and, as stated above, executive deficits are a hallmark of cognitive dysfunction in PD [44,45]. As attention is also a domain frequently affected in PD, the fact that all three subtests which are assigned to the attention domain in the MoCA (digit span, target tapping, serial subtraction) only receive a low weight is unexpected. Remarkably, while only 10 out of our 25 PD patients with cognitive impairment in study 1 failed one of the MoCA attention tests, 17 patients showed deficits on at least one attention test in the elaborate neuropsychological test battery (digit span forward, digit span backward). One possible explanation for this discrepancy is that the MoCA subtests do not validly measure the underlying construct of attention or at least are not sensitive enough. In fact, the difficulty level of these tasks in the MoCA is low. For example, the digit span backwards only requires the repetition of three numbers in reverse order which is comparably simple. This aspect needs further investigation.

Interestingly, the three MoCA subtests that have been suggested to lack discriminatory value in previous research including a PD sample [23] also receive the lowest weight in our suggested scoring: the target tapping, the naming, and the orientation tasks. Finally, the two specific subtests with the highest predictive accuracy (alternating trail making and clock-drawing) are also in line with previous studies examining the diagnostic accuracies of MoCA subtests in a non-PD population [46,47]. This further supports our conclusions about the diagnostic utility of the individual subtests and suggests that the proposed new scoring algorithm may also be valuable to detect cognitive deficits in other patient groups. Finally, it should be noted that despite the high accuracy of the alternating trail making task in the MoCA, the TMT of the CERAD test battery did not differentiate between groups. Although speculative, one could hypothesize that the criterion for correct or incorrect in the MoCA is a qualitative one based on mistakes, whereas the TMT B/A index in the CERAD measures the extra amount of time a patient needs to accomplish the executive task B in comparison to the more simple version in task A, and that the qualitative measure is more sensitive. A second possible explanation is that the alternating trail making task is the first task that has to be accomplished in the MoCA so that it might be especially difficult for the patients to adapt themselves to the task. Another simple explanation that has to be taken into account is that the PD-D group was already slow in part A of the TMT because of a generally slowed speed of processing and that the B/A Index was thus given an unjustified high Z-score. However, these aspects need further research.

The present study is not the first one that recognized the necessity to compensate for some weaknesses of the MoCA while at the same time attempting to preserve its strengths.

Koski, Xie, and Finch [48] administered the MoCA to 222 patients who scored at least 20 out of 30 on the MMSE and analyzed the data with a unidimensional Rasch model. They concluded that many items in the MoCA (the orientation items city, place, year, and month, naming a lion and a camel, and the digit span forward) were found to be too easy for the population for which it was intended and that therefore, many non-demented patients had to answer “unnecessary” items for which the probability of a correct response is close to 100%. They suggested that dropping easy items and constructing additional difficult items would improve the precision of the scale for estimating cognitive ability in non-demented patients—the population for which it was originally designed. They further recommended that if the MoCA is primarily used for patients with milder cognitive dysfunction, dropping the easier items may have the potential to shorten the length of the test without losing any information. The approach of the current study constitutes a practical and valuable alternative to the suggestion of Koski et al. [48] in that it leaves the content of the subtests unchanged, while at the same time decreasing the influence of items which might show a ceiling effect in patients which are only mildly impaired. In another study, Koski, Xie, and Konsztowicz [49] demonstrated that a more accurate estimate of cognitive ability may be achieved by combining information from the MoCA and the MMSE. They suggested that adding information from the MMSE (three-word repetition and recall, copy pentagons, repeat sentence, and write sentence) to the MoCA would reduce measurement error by 13.8% and improve measurement of cognition in the upper levels of ability. However, the algorithm they developed was only intended for quantitative comparison of patients or tracking change over time and not for screening purposes.

Following the discussion about different cutoff scores in the section above, another approach to improve the diagnostic accuracy of the MoCA attempted by some research groups was to change the threshold for cognitive impairment. Larner [10] and Waldron-Perrine and Axelrod [50] suggested a lower cutoff score (< 21/30) than that suggested by the authors of the MoCA (< 26/30) [11]. Luis et al. [51] administered the MoCA to patients with AD, MCI, and normal cognition and the results of their ROC analysis showed that a cutoff score of 23/30 might be superior. Damian et al. [46] empirically examined the threshold scores of the MoCA and suggested that while the original cutoff of 26/30 may be optimal for screening in primary care, a cutoff score of 24/30 would improve the predictive value of the MoCA in settings where the probability of cognitive impairment is comparably high (20% prevalence of MCI or dementia). The discussion concerning different cutoffs for the detection of cognitive impairment is important, yet it does not solve the problem that the subtests that are especially relevant for the detection of dysfunction receive an insufficient number of points and that their diagnostic power should be exploited. On the contrary, lowering the cutoff score for cognitive impairment could even exacerbate the problem in that the “easy” items with a high number of points would have an even stronger influence on the classification of the cognitive state. This may result in an increased number of false negatives which would ultimately lead to a decreased sensitivity of the test.

Several limitations need to be considered when interpreting the findings of the current study. First, the sample size, especially in study 2, was comparably small. This problem generally increases the probability that samples may be selected and non-representative. However, despite this shortcoming, the results were broadly similar in both studies, so that the data sets validate each other. Furthermore, the patients were recruited consecutively, possibly reducing the probability of selectiveness. Nevertheless, further studies with larger patient samples are urgently needed to confirm the higher diagnostic value of the new MoCA scoring algorithm. A second limitation of the study concerns the method of weighting the subtests and the difference of points in relation to the difference in AUCs. In order to achieve a meaningful solution concerning the weighting, a mathematical operation was necessary in form of creating equally sized groups based on the AUCs of the subtests, instead of directly deducing the points from the AUCs. With a bigger sample size and thereby a possibly broader range of AUCs, this intermediate step might prove unnecessary and could be omitted, and a more direct and precise weight allocation might be achieved. This might even further enhance diagnostic accuracy of the MoCA. Again, future studies might face this task. Furthermore, it has to be considered that depression may have affected performance in the MoCA, as depression was not an exclusion criterion in our patients. In fact, mean scores on the GDS-15 were above the cutoff of 4/5 points on depressive symptoms [52] in the PD-D group in both samples. However, mean scores were still relatively low with 7.7 and 5.9. Furthermore, as depression is very common among patients with PD [53], and is related to severity of cognitive symptoms [54], this pattern was expected. Most importantly, cognitive screening instruments are suitable to detect cognitive dysfunction, but not to clarify the cause of cognitive symptoms or differentiate between primary cognitive symptoms and deficits caused by depressive symptoms. Thus, depressive symptoms were not considered in the development of the weighting procedure which was explicitly based on a typical sample of PD patients. A more general limitation is that the MoCA only offers one cutoff score for “impaired” versus “not impaired” and different levels of impairment are neglected. As we intended to keep the test as close to the original version as possible, we did not suggest additional cutoff scores for a more differentiated evaluation of cognitive functions and forwent a ROC analysis for the different PD groups (PD-MCI and PD-D). However, it would be a valuable approach for future studies with large sample sizes of patients with different levels of cognitive impairment. It should also be noted that there was a relatively large age range of PD patients in both study 1 and 2 which was not controlled for, and that it cannot be ruled out that age may have confounded the cognitive measures. However, both samples were consecutively recruited clinical samples of PD patients, and it can thus be assumed that they represent a typical group of patients presenting in neurological settings. Finally, it should be mentioned that alternative approaches to determine the relative importance and discriminating power of the individual MoCA items exist, such as discriminant function analysis and logistic regression. It would be valuable to use and compare one of the alternative approaches in further studies.

The results of our study suggest that weighting the MoCA subtests according to their respective diagnostic values may optimize diagnostic accuracy in the assessment of cognitive impairment of PD patients. Further studies with larger samples sizes of PD patients to evaluate the new scoring system are needed. One of the strengths of the proposed approach lies in improving the detection of patients with milder forms of cognitive impairment, who are the ideal target group for interventions aimed at preventing or slowing the onset of dementia. Future interventions are in fact likely to yield the greatest benefit if initiated in an early phase, when cognitive deficits are mild [55]. Next to pharmacological therapy, non-pharmacological treatments to enhance cognition in PD or to prevent a further deterioration of functions of cognitively impaired PD patients have attracted increasing interest with promising results e.g. for cognitive training and physical exercise [56]. Precise evaluation of cognitive dysfunction and its stabilization or enhancement induced by interventions is crucial, and a version of the MoCA allowing a more precise scoring may contribute to that.

As already mentioned above, another strength of the approach adopted in the current study is that, in contrast to other attempts at correcting the limitations of the MoCA by adding or deleting subtests, the item compilation of this established instrument is not changed and that only an additional scoring table is necessary. This has several advantages: No administrative burden will be placed on clinicians and there is also no need for the clinician to acquire the rules and instructions of an additional/new test. Since testing costs time and money, there is also a potential reduction with regard to financial and time investment. In addition, it would be possible to compare MoCA results to previous studies, because the raw values remain the same. Furthermore, it would theoretically be possible to develop different weights and scoring systems for different patient groups. More specifically, the same test could be used for different patient groups and different scoring procedures could be applied. Finally, a more general advantage is that the items of the MoCA were carefully chosen by the developers of the MoCA [11] and only the scoring system is insufficiently elaborate.

On a final note, it should be said that even if an optimally weighted screening test is created in this way, it is unlikely that it will ever approach the reliability and validity of a well-established and psychometrically sound neuropsychological test battery. Nevertheless, screening procedures have high value as a time-economic, easy to use tool for a first step in detecting cognitive impairment in clinical practice. For PD patients (and also patients with other clinical conditions), they are embedded as a crucial element in the diagnostic process. More specifically, they serve Level I diagnosis of PD-MCI according to Litvan et al. [8] and Level I diagnosis of PD-D according to Dubois et al. [57].

## Supporting Information

S1 Fig Case report of a PD-MCI patient. (PDF)

S1 Table Z-Scores of neuropsychological test battery for all cognitive groups in study 1. (PDF)

S2 Table Z-Scores of neuropsychological test battery for all cognitive groups in study 2. (PDF)
