# Supplementary material for: Screening for Cognitive Impairment in Parkinson's Disease: Improving the Diagnostic Utility of the MoCA through Subtest Weighting
Source: PLoS One. 2016 Jul 20;11(7):e0159318. doi: 10.1371/journal.pone.0159318 (PMC4954721; doi:10.1371/journal.pone.0159318)

S1 Fig: Case report of a PD-MCI patient

Our patient was a 78 year old female patient who was diagnosed with PD ten years prior to our examination at the age of 68 years. She had 19 years of formal education. In the elaborate neuropsychological test battery, she showed impairment in executive functions, memory, and attention. She had no dysfunction of activities of daily living (ADL) that could be assigned to her cognitive impairment. She was classified as having PD-MCI according to Movement Disorder Society (MDS) Task Force Level II criteria (Litvan et al., 2012). On the basis of the new scoring algorithm for the MoCA as indicated in Table 2, the MoCA subtests of our patient were re-weighted and the classification of her cognitive status changed:

MoCA Subtest	Original (raw) points (received/maximum)	New transformed points (received/maximum)
Alternating Trail Making	0/1	0/4
Cube copy	0/1	0/3
Clock-drawing	3/3	5/5
Animal Naming	3/3	1/1
Digit Span	2/2	1/1
Target tapping	1/1	1/1
Serial subtraction	3/3	1/1
Repetition	2/2	3/3
Verbal fluency	1/1	3/3
Abstraction	2/2	1/1
Learning	0/0	2/3
Memory	3/5	2/3
Orientation	6/6	1/1
**Total Score**	**26**	**21**

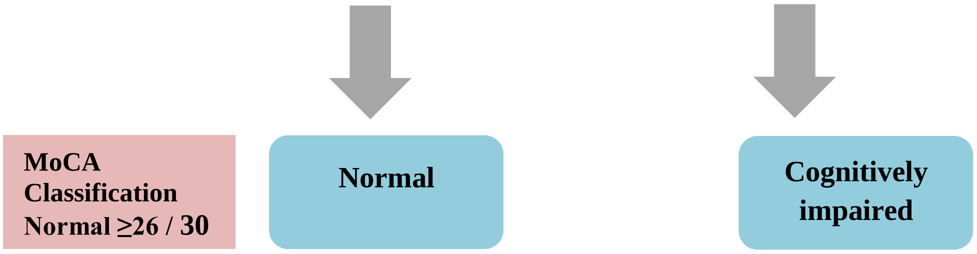

Supplement: S1 Fig — (PDF) [file pone.0159318.s001.pdf]
