# Supplementary material for: Screening for Cognitive Impairment in Parkinson's Disease: Improving the Diagnostic Utility of the MoCA through Subtest Weighting
Source: PLoS One. 2016 Jul 20;11(7):e0159318. doi: 10.1371/journal.pone.0159318 (PMC4954721; doi:10.1371/journal.pone.0159318)
Supplement: S1 Table — (PDF) [file pone.0159318.s002.pdf]

S1 Table. Z-Scores of neuropsychological test battery for all cognitive groups in study 1.

		PD-N (n= 15)	PD-MCI (n= 14)	PD-D (n= 11)	*p*-value	Effect size *r*	PD-cognitively impaired (PD-MCI&PD-D; n=25)	*p*-value (PD-N vs. PD-cognitively impaired)	Effect size *r*
Verbal comprehension [a,b]	Mean (SD)	18.0 (0.0)	17.8 (0.8)	17.14 (1.464)			17.57 (1.08)		
	Median (IQR)	18.0 (18-18)	18.0 (18.0-18.0)	18.0 (15.0-18.0)	.082	.38	18.0 (18.0-18.0)	.485	.25
Immediate Recall [c]	Mean (SD)	0.06 (0.62)	-1.42 (1.28)	-1.9 (1.02)	<.001 [e]	.64	-1.62 (1.18)	<.001	.63
	Median (IQR)	0.02 (-0.44-0.31)	-1.62 (-2.1-(-0.42))	-1.85 (-2.69-(-1.16))			-1.62 (-2.49-(-0.86))		
Delayed Recall [c]	Mean (SD)	-0.17 (0.67)	-0.99 (1.29)	-1.69 (1.06)	.006 [f]	.51	-1.25 (1.24)	.004	.46
	Median (IQR)	-0.09 (-0.6-0.29)	-1.31 (-1.71-0.27)	-1.88 (-2.42-(-1.04))			-1.54 (-2.1-(-0.1))		
Semantic fluency [c]	Mean (SD)	-0.21 (1.1)	-0.77 (1.19)	-1.78 (1.0)	.004 [f]	.51	-1.21 (1.2)	.011	.40
	Median (IQR)	-0.34 (-0.77-0.21)	-0.50 (-1.7-0.18)	-1.55 (-2.1-(-1.25))			-1.27 (-2.05-(-0.28))		
Trail Making Test B/A [c]	Mean (SD)	0.07 (0.86)	-0.62 (0.78)	-0.67 (0.76)	.057	.40	-0.64 (0.76)	.016	.41
	Median (IQR)	0.02 (-0.74-0.62)	-0.83 (-1.07-(-0.23))	-0.86 (-1.51-0.22)			-0.83 (-1.09-(-0.17))		
Constructional praxis [b]	Mean (SD)	0.2 (1.2)	-1.32 (1.54)	-1.43 (2.08)			-1.37 (1.75)		
	Median (IQR)	0.6 (0.24-0.89)	-1.54 (-2.85-0.44)	-1.56 (-3.37-0.61)	.027	.44	-1.57 (-2.86-0.47)	.006	.43
Boston Naming Test [b]	Mean (SD)	0.62 (0.51)	0.01 (0.89)	-1.01 (1.0)			-0.41 (1.05)		
	Median (IQR)	0.63 (0.41-0.87)	0.15 (-0.81-0.85)	-0.9 (-1.36-(-0.52))	.001 [g]	.63	-0.73 (-0.93-0.78)	.004	.45
Digit span backwards [b d]	Mean (SD)	41.53 (29.93)	37.9 (29.7)	40.0 (26.83)			38.79 (27.92)		
	Median (IQR)	27 (13-53)	32.5 (11.75-59.25)	40 (13-67)	.918	.07	32.5 (13.0-63.5)	.809	.04
Block span forwards [b d]	Mean (SD)	54.33 (37.78)	30.1 (26.7)	-15.1 (30.4)			24.68 (28.19)		
	Median (IQR)	57.0 (8-95)	28.0 (6.5-58)	4.5 (2-8)	.013	.49	7.5 (2.0-57.25)	.008	.43
Mental rotation [c d]	Mean (SD)	70.8 (19.22)	57.23 (19.3)	50.0 (36.6)	.127	.35	54.93 (25.15)	.052	.34
	Median (IQR)	72.0 (55-86)	60.0 (41.5-71)	59.5 (6.93-76.75)			60.0 (40.0-70.0)		

SD, Standard deviation; IQR, Interquartile range

[a] Raw values are shown.
[b] The Kruskal-Wallis test with Bonferroni adjusted alpha-levels of 0.005 per test was used for variables that were not normally distributed. P-values are shown for the median. Groups were compared using the Mann-Whitney U test with Bonferroni adjusted alpha-levels of 0.0017 per test. Means and standard deviations are also reported for comparison purposes. The effect size *r* was obtained by computing partial eta-squared on the ranked scores and taking the square root.
[c] A univariate Between-Subjects ANOVA with Bonferroni adjusted alpha-levels of 0.005 were used for variables that were normally distributed. P-values are shown for the mean. Post-hoc comparisons were conducted using Bonferroni corrections. Median and interquartile range are also reported for comparison purposes.
[d] Percentile ranks are shown.
[e] Pairwise comparisons showed that PD-N>PD-MCI; PD-MCI=PD-D; PD-N>PD-D.
[f] Pairwise comparisons showed that PD-N=PD-MCI=PD-D; PD-N>PD-D.
[g] Pairwise comparisons showed PD-N=PD-MCI; PD-MCI>PD-D; PD-N>PD-D.
