# Supplementary material for: Screening for Cognitive Impairment in Parkinson's Disease: Improving the Diagnostic Utility of the MoCA through Subtest Weighting
Source: PLoS One. 2016 Jul 20;11(7):e0159318. doi: 10.1371/journal.pone.0159318 (PMC4954721; doi:10.1371/journal.pone.0159318)
Supplement: S2 Table — (PDF) [file pone.0159318.s003.pdf]

S2 Table. Z-Scores of neuropsychological test battery for all cognitive groups in study 2.

		PD-N (n= 8)	PD-MCI (n= 10)	PD-D (n= 6)	*p*-value	Effect size r	PD-cognitively impaired (PD-MCI&PD-D; n=16)	*p*-value (PD-N vs. PD-cognitively impaired)	Effect size r
Verbal comprehension [a,b]	Mean (SD)	17.9 (0.4)	18.0 (0.0)	17 (1.6)			17.7 (1.0)		
	Median (IQR)	18 (18.0-18.0)	18.0 (18.0-18.0)	18.0 (15.0-18.0)	.138	.41	18.0 (18.0-18.0)	.976	.02
Immediate Recall [c]	Mean (SD)	0.07 (0.98)	-1.28 (1.15)	-3.24 (1.14)	<.001 [e]	.77	-2.02 (1.47)	.002	.61
	Median (IQR)	0.26 (-1.03-1.04)	-1.7 (-2.0-(-0.4))	-3.44 (-3.91-(-2.18))			-1.95 (-3.18- (-1.25))		
Delayed Recall [c]	Mean (SD)	-0.43 (0.9)	-1.47 (0.59)	-3.17 (0.89)	<.001 [e]	.82	-2.11 (1.09)	<.001	.62
	Median (IQR)	-0.35 (-0.76-0.39)	-1.52 (-1.78-(-1.25))	-3.21 (-4.0-(-2.46)			-1.78 (-2.68-(-1.51)		
Semantic fluency [b]	Mean (SD)	0.65 (0.63)	-1.04 (1.15)	-1.71 (0.72)			-1.29 (1.04)		
	Median (IQR)	0.74 (0.13-1.02)	-1.7 (-1.93-0.06)	-1.92 (-2.2-(-1.33))	.002 [f]	.73	-1.7 (-2.12-(-0.35)	<.001	.68
Trail Making Test B/A [c]	Mean (SD)	0.04 (0.86)	-0.34 (1.06)	0.69 (0.93)	.147	.41	.045 (1.12)	.651	.20
	Median (IQR)	0.13 (-0.78-0.78)	-0.72 (-1.1-0.35)	1.05 (-0.19-1.36)			-0.12 (-0.96-1.24)		
Constructional praxis [b]	Mean (SD)	0.32 (0.65)	-0.34 (1.43)	-1.54 (1.88)			-0.79 (1.66)		
	Median (IQR)	0.51 (-0.13-0.72)	0.52 (-1.68-0.7)	-1.2 (-3.19-0.52)	.165	.40	-0.43 (-2.59-0.66)	.214	.26
Boston Naming Test [b]	Mean (SD)	0.72 (0.66)	-0.01 (0.97)	-1.23 (1.67)					
	Median (IQR)	0.91 (0.41-1.25)	0.18 (-0.92-0.73)	-1.53 (-2.69-0.38)	.035	.54	-0.36 (-1.43-0.69)	.023	.46
Digit span backwards [b d]	Mean (SD)	45.38 (25.65)	41.6 (33.66)	18.2 (19.72)			33.8 (31.14)		
	Median (IQR)	45.5 (19.25-63.5)	43.5 (11.0-71.5)	12.0 (6.5-33.0)	.158	.41	13.0 (8.0-53.0)	.238	.25
Block span forwards [b d]	Mean (SD)	52.13 (22.92)	29.8 (23.97)	27.83 (24.9)			29.06 (23.5)		
	Median (IQR)	57.0 (27.25-73.0)	28.0 (6.0-52.0)	20.5 (6.5-58.0)	.237	.35	28.0 (8.0-56.0)	.093	.35
Mental rotation [c d]	Mean (SD)	49.13 (27.79)	37.67 (19.1)	22.6 (18.66)	.147	.43	32.29 (19.7)	.112	.35
	Median (IQR)	62.5 (17.0-73.5)	37.0 (23.0-52.5)	11.0 (9.0-42.0)			32.0 (11.0-50.0)		

SD, Standard deviation; IQR, Interquartile range

[a] Raw values are shown.
[b] The Kruskal-Wallis test with Bonferroni adjusted alpha-levels of 0.005 per test was used for variables that were not normally distributed. P-values are shown for the median. Groups were compared using the Mann-Whitney U test with Bonferroni adjusted alpha-levels of 0.0017 per test. Means and standard deviations are also reported for comparison purposes. The effect size *r* was obtained by computing partial eta-squared on the ranked scores and taking the square root.
[c] A univariate Between-Subjects ANOVA with Bonferroni adjusted alpha-levels of 0.005 were used for variables that were normally distributed. P-values are shown for the mean. Post-hoc comparisons were conducted using Bonferroni corrections. Median and interquartile range are also reported for comparison purposes.
[d] Percentile ranks are shown.
[e] Pairwise comparisons showed that PD-N=PD-MCI; PD-MCI>PD-D; PD-N>PD-D.
[f] Pairwise comparisons showed that PD-N>PD-MCI; PD-MCI=PD-D; PD-N>PD-D.
